# Multi-Spectroscopic and Molecular Simulation Approaches to Characterize the Intercalation Binding of 1-Naphthaleneacetic Acid With Calf Thymus DNA

**DOI:** 10.3389/ftox.2021.620501

**Published:** 2021-04-28

**Authors:** Xing Hu, Xiaoqiao Luo, Zhisheng Zhou, Rui Wang, Yaqin Hu, Guimei Zhang, Guowen Zhang

**Affiliations:** State Key Laboratory of Food Science and Technology, Nanchang University, Nanchang, China

**Keywords:** 1–naphthaleneacetic acid, calf thymus DNA, spectroscopic analysis, intercalation, molecular docking

## Abstract

1–Naphthaleneacetic acid (NAA), having high-quality biological activity and great yield-increasing potential in agricultural production, is a broad-spectrum plant growth regulator. Although NAA is of low toxicity, it can affect the balance of the human metabolism and damage the body if it is used in high quantity for a long time. In this study, the interaction of NAA with calf thymus DNA (ctDNA) was investigated under simulated human physiological acidity (pH 7.4) using fluorescence, ultraviolet-visible absorption, and circular dichroism spectroscopy combined with viscosity measurements and molecular simulation techniques. The quenching of the endogenous fluorescence of NAA by ctDNA, observed in the fluorescence spectrum experiment, was a mixed quenching process that mainly resulted from the formation of the NAA–ctDNA complex. NAA mainly interacted with ctDNA through hydrophobic interaction, and the binding constant and quenching constant at room temperature (298 K) were 0.60 × 10^5^ L mol^−1^ and 1.58 × 10^4^ L mol^−1^, respectively. Moreover, the intercalation mode between NAA and ctDNA was verified in the analysis of melting point, KI measurements, and the viscosity of ctDNA. The results were confirmed by molecular simulation, and it showed that NAA was enriched near the C–G base of ctDNA. As shown in circular dichroism spectra, the positive peak intensity of ctDNA intensified along with a certain degree of redshift, while the negative peak intensity decreased after binding with NAA, suggesting that the binding of NAA induced the transformation of the secondary structure of ctDNA from B-form to A-form. These researches will help to understand the hazards of NAA to the human body more comprehensively and concretely, to better guide the use of NAA in industry and agriculture.

## Introduction

DNA is the carrier of biological genetic information, regulating protein synthesis through transcription and translation, it controls the growth, development, and apoptosis of organisms (Wang et al., 2019). At the same time, DNA is also the primary target of many exogenous small molecules, such as drugs, fatty acids, and metal ions complexes, etc. (Qais et al., 2017; Liu et al., 2019; Zianna et al., 2019). However, the covalent or non-covalent binding between toxic molecules and DNA affects DNA structure, and can even affect its transcription and translation (Roy et al., 2015). Attacks on DNA by exogenous substances are also closely related to the occurrence of abnormal phenomena such as tumors and hereditary diseases (Wang and Groopman, 1999; Tubbs and Nussenzweig, 2017). It is therefore of far-reaching significance to research the interaction of exogenous toxic small molecules with DNA. Better knowledge of this interaction will help the public to understand the side effects of the substance and provide a theoretical foundation for the supervision and application of the substance.

**Figure 1:**
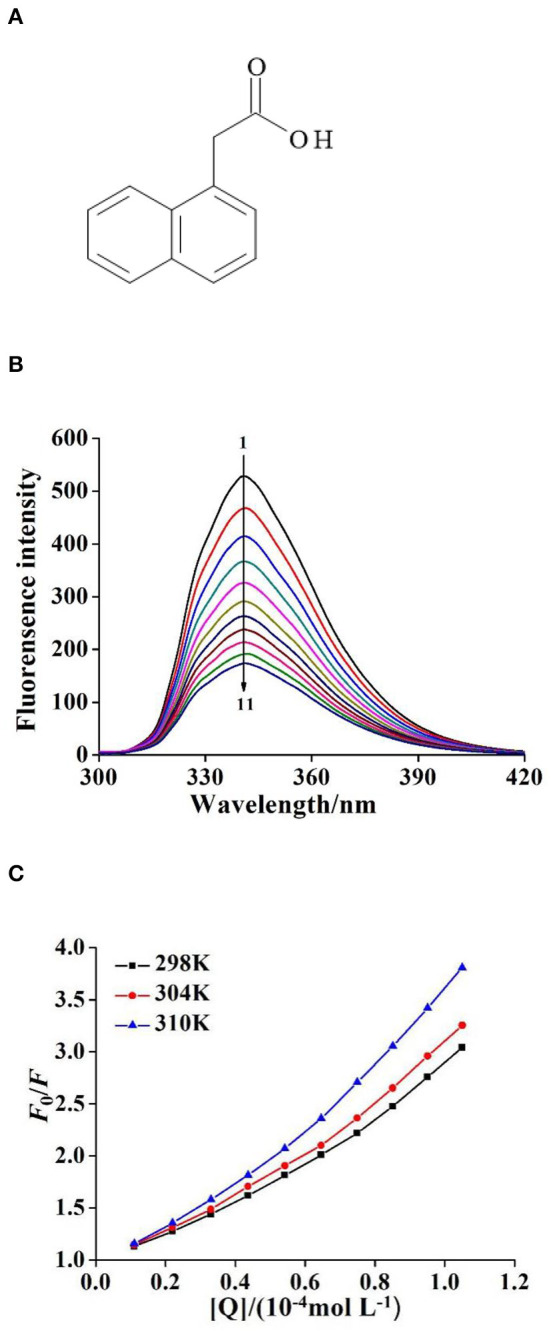
**(A)** The structure of 1–Naphthaleneacetic acid (NAA). **(B)** The quenching fluorescence spectra of NAA–ctDNA system (T = 298*K*, λ_ex_ = 280*nm*, λ_em_= 341 nm). *c*(NAA) = 2.50 × 10^−6^ mol L^−1^, *c*(ctDNA) = 0, 0.11, 0.22, …, 1.05 × 10^−4^ mol L^−1^ for curves 1 → 11, respectively. **(C)** The Stern–Volmer plots for the fluorescence quenching of NAA by ctDNA at unlike temperatures (298, 304, and 310 K).

Plant growth regulators (PGRs) are synthetic pesticides with similar effects to natural plant hormones (Rademacher, 2015). They are widely used in agricultural production to regulate plant growth and development (Fahad et al., 2016). In general, PGRs are conducive to plant growth and development at lower concentrations and inhibit plant growth and development at higher concentrations (Vandenbussche and Van Der Straeten, 2012; Jamwal et al., 2018). In agricultural production, appropriate concentrations can be selected according to production needs. Naphthaleneacetic acid (see Figure 1A) is one of the PGRs. It has the functions of promoting plant growth, changing the ratio of male to female flowers, increasing fruit setting, and preventing fruit drop, etc. (Rademacher, 2015). It is divided into two configurations, α and β types. Because the activity of α-type is stronger, the use of α-Naphthaleneacetic acid (α-NAA) is more common. NAA plays an indispensable role in agricultural production. However, because of its benefits, some people often neglect to remember that NAA is a pesticide and they increase the concentration casually, resulting in high residues in agricultural products and soil (Li et al., 2020). These high-residue NAAs not only cause pollution to the environment, but they may also even enter the human body through diet and endanger human health.

By examining the effect of a number of plant growth regulators on the thermal stability of DNA isolated from pea seedlings, Elchanan (1971) summarized three types of interactions with DNA: (1) Indole-3-acetic acid, Gibberellic acid, 2,4-Dichlorophenoxy acetic acid, and 2,4,5-Trichlorophenoxy acetic acid destabilized portions of the native DNA molecules. (2) 6-Furfurylaminopurine and 6-Benzylaminopurine stabilized DNA to a certain extent at low ionic strength. (3) Indole-3-propionic acid and Indole-3-butyric acid in different concentrations showed opposite effects on DNA. Zhu et al. (2021) reported Indole-3-acetic acid bound to the minor groove region rich in G–C base-pairs in ctDNA, which disturbed the structure of ctDNA but did not induce DNA damage. Studies have shown that NAA has a stimulating effect on sensitive skin and mucous membranes in the human eyes and respiratory tract, etc (Ilktaç et al., 2019). The oral LD_50_ of NAA for rats, mice, and mammals is 1, 0.743, and 1 g/kg, respectively (Nikolelis et al., 2008). However, studies of the interactions between plant growth regulators and DNA are still rare. There are few reports on the toxicity and mechanism of NAA on humans and animals at home and abroad. The mechanisms of NAA harm to the human body are still inconclusive.

In this study, under simulated physiological conditions (pH 7.4), a variety of spectroscopic means, including UV–vis absorption, fluorescence, and circular dichroism (CD) spectroscopy along with molecular modeling and other methods, were used to investigate the binding properties of NAA to calf thymus DNA (as ctDNA model), to further reveal the mechanism of action and potential damage NAA causes to ctDNA. This research furthers understanding of the binding mechanism of NAA with ctDNA and the toxic effects of NAA, providing new insights for better use of NAA in the future.

## Experimental Materials and Methods

### Materials

NAA (analytical purity, 98%) was purchased from Aladdin Reagent Company (Shanghai, China) and prepared with anhydrous ethanol as a reserve solution, with a concentration of 5.0 × 10^−3^ mol L^−1^ for cryogenic and light-proof preservation. The preparation method of ctDNA (Sigma Chemical Reagent Co., Ltd., USA) reserve solution is as follows: dissolve the appropriate amount of ctDNA in 0.1 mol L^−1^ NaCl solution and shake it gently until it dissolves. Then determine the ratio of ultraviolet absorbance of ctDNA reserve solution at 260 and 280 nm to 1.91 (*A*_260_/*A*_280_ = 1.91>1.8), indicating that the purity of ctDNA concentrate meets the requirements (Zhou et al., 2019). Finally, the absorbance value of ctDNA solution at 260 nm was determined, and the concentration in the reserve solution was calculated to be 3.22 × 10^−3^ mol L^−1^ by using molar absorbance coefficient ε_260_ = 6,600 L mol^−1^ cm^−1^. EB (Sigma Chemical Reagent Co., Ltd., USA) prepared a reserve solution with a concentration of 5.83 × 10^−3^ mol L^−1^ from ultrapure water. The buffer solution was 0.05 mol L^−1^ Tris–HCl (pH 7.4). The other chemicals used in the experiment were analytically pure. The experimental water was super pure water from the Milli-Q water purification system (Millipore, Bedford, MA, USA). All reserve liquids were stored in 0~4°C refrigerator.

### Methods and Equipment

#### Fluorescence Spectrometry

NAA has many conjugated structures, so it can produce fluorescence under appropriate ultraviolet irradiation. Using the fluorescence quenching effect of ctDNA on NAA, the binding between ctDNA and NAA can be preliminarily judged. Using 280 nm as excitation wavelength, the slit width of excitation wavelength and emission wavelength was 2.5 nm. The emission spectra of the NAA–ctDNA system at 298, 304, and 310 K at different temperatures are determined by fluorescence spectrophotometer (F−7000, Hitachi, Japan) in the range of 300~500 nm.

Preparing the ctDNA solution (1.85 × 10^−4^ mol L^−1^) and NAA–ctDNA solution (*c*(ctDNA) = 1.85 × 10^−4^ mol L^−1^, *c*(NAA) = 5.00 × 10^−5^ mol L^−1^). The fluorescence quenching of NAA–ctDNA complexes and individual NAA solution with different concentration of KI were measured (λ_ex_ = 280 nm, Δλ_ex_ = Δλ_em_ = 2.5 nm). Then, the quenching constants of KI on NAA and NAA–ctDNA system were calculated in light of the Stern–Volmer equation.

For deducting the effect of solution reabsorption, all fluorescence data selected for calculation were corrected with the following equation (Zhang et al., 2014):


(1) $$\begin{array}{l} F_{c}=F_{m}e^{\left(A_{1}+A_{2}\right)/2} \end{array}$$

Wherein *F*_c_ and *F*_m_ are the corrected and measured fluorescence intensities, respectively, and *A*_1_ and *A*_2_ are the UV–vis absorbances of the quencher at the excitation and emission wavelengths, respectively (TU1901, Preliminary, Beijing).

#### Viscosity Experiments

Viscosity measurements were carried out according to the literature and were slightly modified based on existing research (Ahmad et al., 2016). The Ubbelohde viscometer (inner diameter 0.7~0.8 mm, Shanghai Qianfeng Rubber and Glass Co., Ltd., Shanghai, China) was placed in a constant temperature water bath at 25°C. The time required for the NAA–ctDNA system and EB–ctDNA system to flow the capillary was measured, respectively. Every sample was measured five times in parallel and confirmed by a digital stopwatch. The time (*t*) required for a fixed sample to flow the capillary was thus determined. Then the relative viscosity was calculated according to the formula η = (*t*–*t*_0_)/*t*_0_, in which η and *t* were the relative viscosity and the flow time of ctDNA solution underneath different concentrations of NAA or EB, respectively, and *t*_0_ was the time required for Tris–HCl buffer solution to flow the capillary. Finally, the results were plotted with (η/η_0_)^1/3^ molar ratio [[NAA]/[ctDNA]] or [[EB]/[ctDNA]], where η_0_ was the relative viscosity of ctDNA in buffer solution.

#### DNA Melting Point Experiment

To determine the melting temperature of ctDNA and NAA–ctDNA complexes, a single ctDNA solution (1.85 × 10^−4^ mol L^−1^) and an NAA–ctDNA mixed system (in which the concentration of ctDNA and NAA were 1.85 × 10^−5^ mol L^−1^ and 5.00 × 10^−5^ mol L^−1^, respectively) were prepared. Then the absorbance of the two solutions at 260 nm was measured at intervals of 5°C in the temperature range of 20~100°C. The melting point (*T*_m_) of DNA is calculated by the following formula: *f*_ss_ = (*A*–*A*_0_)/(*A*_f_-*A*_0_), where *A, A*_0_, and $A_{{\text{AE}}^{\prime }}$ are the absorbance of the solution at different temperatures, original and final temperatures, respectively, and then draw the curve of Æ'_ss_ to temperature (T). When *f*_ss_ = 0.5, the temperature is the melting point temperature (Ahmad and Ahmad, 2015).

#### Ion Effect Experiment

Prepared the solution of ctDNA and NAA–ctDNA complex, respectively, and let the complex solution stood at room temperature for 8 h. Then, added NaCl solution (0~1.64 × 10^−2^ mol L^−1^) to the above solution, and measured the absorption spectra of each solution, respectively.

#### Circular Dichroism Determination

CD spectra were measured using a MOS 450 CD spectrometer (Bio–Logic, Claix, France). The ctDNA concentration was fixed at 6.44 × 10^−4^ mol L^−1^, and a mixture of NAA and ctDNA in different molar ratios was prepared [r = [NAA]/[ctDNA] = 0, 1/20 and 1/10]. Then, the CD spectra of ctDNA and NAA mixed solutions in the wavelength range of 220~320 nm were scanned. The background was subtracted with Tris–HCl buffer solution before scanning.

#### Molecular Simulation

The most favorable binding conformation of NAA–ctDNA was estimated by AutoDock 4.2 software. The crystal structure of the simulated ctDNA (ID: 8BNA) and the 3D structure of NAA were obtained by Protein Data Bank (PDB) (http://www.rcsb.org/pdb) and Chem3D Ultra 8.0 software, respectively. Before docking, the PDB files of ctDNA were processed, water was removed and hydrogenated, and Gasteiger was added by the AutoDock tool (ADT) (Zhang et al., 2017). Then, the molecular simulation was carried out based on the Lamarckian genetic algorithm (LGA), the running space was set to 120 Å × 120 Å × 120 Å. When starting the molecular simulation, the structure of ctDNA is fixed, the ligands are allowed to move in the designated region, and docking times were 100. Finally, the optimal binding conformation of the NAA–ctDNA complex was analyzed by considering the optimal docking results of docking times and docking energy selection. The root mean square deviation (RMSD) of 2.0 was used for analyzing the docking results.

#### Statistical Analysis

All measurements were carried out three times in parallel. The experimental results were expressed by mean ± standard deviation *(n* = 3), and Origin Pro 8 SR0 (Origin Lab, Northampton, MA) was used to analyze the results by one-way ANOVA. The results were statistically significant when *p* < 0.05.

## Results and Discussion

### Analysis of Fluorescence Quenching Effect of ctDNA on NAA

As shown in Figure 1B, when the excitation wavelength was 280 nm, the maximum emission peak of NAA was 341 nm. With the continuous addition of ctDNA solution, the fluorescence intensity of NAA showed a uniform downward trend. Based on forming a complex or depending on the collision between molecules, the classical fluorescence quenching theory divided the fluorescence quenching mechanism into static and dynamic quenching modes (Zhang et al., 2017). Generally, the collision between molecules becomes more intense when the temperature is higher, so the dynamic quenching constant increases with the temperature. Conversely, the thermal stability of the complex decreases with the increase of temperature, so the quenching constant decreases. To clarify the fluorescence quenching mechanism between ctDNA and NAA, the fluorescence experimental data of the NAA–ctDNA system were analyzed by Stern–Volmer equation (Ma et al., 2012):


(2) $$\begin{array}{l} \frac{F_{0}}{F}=1+K_{\text{SV}}\left[Q\right]=1+K_{\text{q}}\tau_{0}\left[Q\right] \end{array}$$

where *F*_0_ and *F* are fluorescence intensity before and after adding ctDNA, *K*_SV_ is quenching constant, *K*_q_ is the rate constant of bimolecular quenching process, [Q] is the concentration of ctDNA, and τ_0_ is the average fluorescence lifetime of NAA without ctDNA, which is about 10^−8^ s. The *K*_SV_ of the NAA–ctDNA system increased with increasing temperature (Figure 1C), which indicates that there was a dynamic quenching in the quenching process. However, as shown in Table 1, the value of *K*_q_ was much larger than the maximum diffusion collision quenching constant (2.0 × 10^10^ L mol^−1^ s^−1^), which indicated that the quenching mechanism of the interaction was a mixed quenching process, and static quenching was the mainly quenching mechanism (Geng et al., 2013). The first five points of the Stern–Volmer curve are very linear, but with the increase of the concentration of ctDNA, the concave surface of the curve bends slightly to the y-axis (Figure 1C), which also indicated that the quenching mechanism was probably a mixed quenching mode (Zeng et al., 2018).

**Table 1:** The quenching constants, binding constants, and thermodynamic properties of the interaction between NAA and ctDNA at different temperatures.

***T* (K)**	***K*_**SV**_** **(×10^**4**^ L mol^**−1**^)**	***R* ^**A**^**	***K*_**A**_** **(×10^**5**^ L mol^**−1**^)**	***R* ^**B**^**	**Δ*H*** **(kJ mol^**−1**^)**	**Δ*G*** **(kJ mol^**−1**^)**	**Δ*S*** **(J mol^**−1**^)**
298	1.58	0.9957	0.60	0.9989	54.04	−27.35	273.1
304	1.79	0.9961	1.03	0.9981		−28.99	
310	2.19	0.9968	1.39	0.9998		−30.63	

*R is the correlation coefficient*.

Then the binding constants (*K*_a_) of the interaction were calculated by the modified Stern–Volmer equation (Ma et al., 2012):


(3) $$\begin{array}{l} \frac{F_{0}}{F_{0}-F_{\;}}=\frac{1}{f_{\text{a}}K_{\text{a}}\left[Q\right]}+\frac{1}{f_{\text{a}}} \end{array}$$

The picture of *F*_0_/(*F*_0_-*F*) vs. 1/[Q] was plotted, and the values of *K*_a_ at 298, 304, and 310 K were calculated and demonstrated in Table 1, respectively. As shown in Table 1, the *K*_a_ value increases with an increase in temperature, indicating that the stability of the complex also increased and there was a dynamic quenching. This further demonstrates the mixed quenching mechanism. Furthermore, the *K*_a_ values manifested an order of magnitude of 10^5^ L mol^−1^ at different temperatures, demonstrating that NAA had a strong affinity to ctDNA. Different from intercalation binding, the binding affinity was weak and the *K*_a_ values were on the order of magnitude of 10^4^ or less when a ligand bound to ctDNA in groove mode (Zhu et al., 2021). Hence, the relatively large *K*_a_ values obtained in our study could be due to the intercalation binding between NAA and ctDNA, similar to the result reported by Garbett et al. (2004) that ethidium bromide intercalated into ctDNA and the binding constant was 1.23 × 10^5^ L mol^−1^.

To further clarify the thermodynamic process of the interaction of NAA with ctDNA, the thermodynamic parameters of the interaction are calculated by the following equations (Liu et al., 2013):


(4) $$\begin{array}{l} \text{log }{\text{K}}_{\text{a}}=-\frac{{\Delta H}^{\;}}{2.303RT}+\frac{{\Delta S}^{\;}}{2.303R} \end{array}$$

(5) $$\begin{array}{l} {\Delta G}^{\;}={\Delta H}^{\;}-{T\Delta S}^{\;} \end{array}$$

where *R* is the gas constant and *T* is the experimental temperature (298, 304, and 310 K). Generally, hydrophobic forces, van der Waals forces, hydrogen bonds, and electrostatic forces are the main interaction forces among small molecules and DNA, and the specific interaction force can be ascertained with thermodynamic parameters. Ross and Subramanian (1981) have shown that the main force of reaction was hydrophobic when Δ*S* > 0 and Δ*H* > 0, electrostatic when Δ*H* = 0 or Δ*H* was small and Δ*S* > 0, hydrogen bond and van der Waals force when Δ*H* < 0 and Δ*S* < 0, hydrogen bond and hydrophobic force when Δ*H* < 0 and Δ*S* > 0. As depicted in Table 1, Δ*G* < 0 indicated that the interaction between ctDNA and NAA was spontaneous. Δ*H* > 0 indicated that the binding of ctDNA and NAA was endothermic. Δ*S* > 0 and Δ*H* > 0 meant that hydrophobic interaction was the main driving force when NAA interacted with ctDNA.

### Viscosity Experimental Analysis

**Figure 2:**
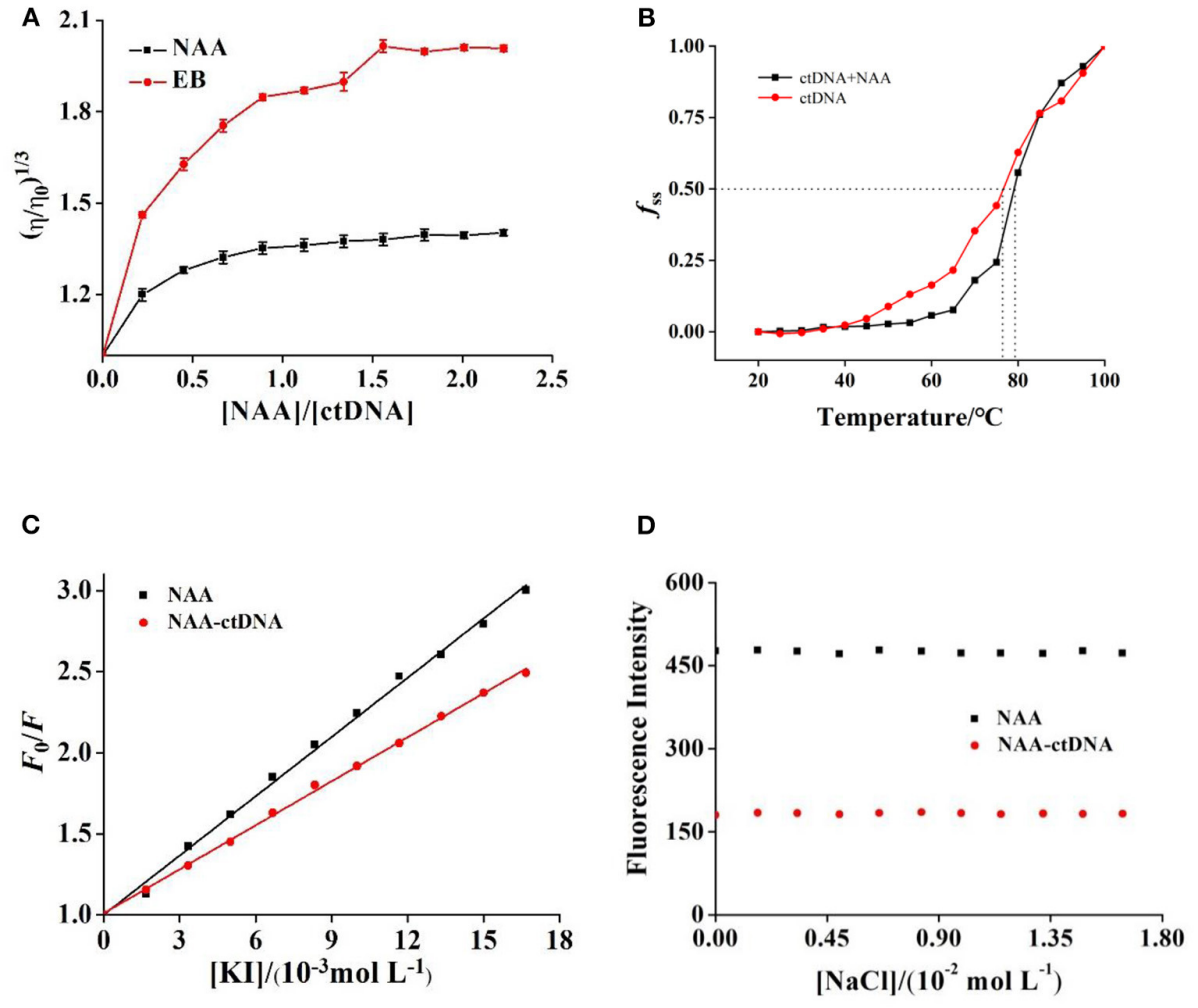
**(A)** Relative viscosity of ctDNA in the presence of different concentrations of NAA or EB at pH 7.4. *c*(ctDNA) = 4.50 × 10^−5^ mol L^−1^. *c*(NAA) = *c*(EB) = 0, 0.99, 2.03, …, 10.04 × 10^−5^ mol L^−1^, respectively. **(B)** Melting curves of ctDNA without and with NAA at pH 7.4. *c*(NAA) = 5.00 × 10^−5^ mol L^−1^ and *c*(ctDNA) = 1.85 × 10^−4^ mol L^−1^. **(C)** The Stern–Volmer plots for the fluorescence quenching of NAA and NAA–ctDNA system by KI. *c*(NAA) = 5.00 × 10^−5^ mol L^−1^ and *c*(ctDNA) = 1.85 × 10^−4^ mol L^−1^. **(D)** Effect of ionic strength on the fluorescence of NAA alone and NAA–ctDNA system. *c*(ctDNA) = 0.85 × 10^−4^ mol L^−1^, *c*(NAA) = 2.50 × 10^−6^ mol L^−1^.

When small molecules are inserted into DNA molecules, to keep space for small molecules, the distance between adjoining base pairs of DNA will increase and the DNA molecules will be lengthened, which will lead to an increase of DNA viscosity (Varna et al., 2018). While non-intercalation modes such as electrostatic binding and groove binding will not change the viscosity of DNA, because their action area is mainly outside the DNA molecules (Hong et al., 2018). Figure 2A shows that the viscosity of ctDNA increases obviously with the increase of NAA concentration and then tends to stabilize gradually, which is similar to the results obtained by EB and pesticide aminocarb (Zhang et al., 2010). The results preliminarily proved that NAA and ctDNA bind by intercalation.

### DNA Melting Point Experiment

DNA denaturation, known as the melting point of DNA, is a process in which double-stranded DNA (ds DNA) are separated into single-stranded DNA (ss DNA) by opening the hydrogen bond between bases (Ali et al., 2018). When the temperature rises slowly to a certain level, the absorbance value of the solution (generally at 260 nm) increases rapidly due to the effect of unwinding. The temperature corresponding to the midpoint of the transition is called melting point temperature (*T*_m_). Targeted small molecule intercalation with DNA will grow the stability of DNA helix structure, but there will be no significant change in the *T*_m_ of DNA in non-intercalation mode (such as groove and electrostatic binding mode).

Figure 2B shows that the *T*_m_ of free ctDNA was about 76.5°C, while that of ctDNA in the presence of NAA was 79.2°C. The *T*_m_ of ctDNA increased after binding with NAA which was consistent with the binding between propyzamide of daunorubicin and ctDNA (Zhang et al., 2015). It can be inferred that the binding of NAA increased the double-stranded structure stability of ctDNA, which proved that the binding of NAA to ctDNA was not groove or electrostatic binding mode.

### KI Experiment

I^−^ is an anionic fluorescence quencher, which repels the negative charge of phosphoric acid on the DNA molecular skeleton. Generally, small molecules bound to DNA by intercalation are encapsulated in the double helix structure, which weakens the effect of I^−^ quenching (Yang et al., 2018). Small molecules bound by electrostatic or groove are exposed to the DNA solution, and their fluorescence is easily quenched by I^−^ (Marina et al., 2018). Therefore, the fluorescence quenching mechanism of small molecules with DNA systems can be compared by I^−^.

The fluorescence quenching constants of I^−^ to NAA and NAA–ctDNA systems were determined, and their values were 1.2 × 10^2^ L mol^−1^ and 0.90 × 10^2^ L mol^−1^, respectively (Figure 2C). It could be concluded that the binding of ctDNA had protected NAA and weakened the fluorescence quenching effect of I^−^ on NAA. These results further confirm that the binding mode of NAA and ctDNA was intercalation.

### Salt Effect Experiment

The ionic strength has a close relation to the electrostatic binding mode. If the ligand binds to ctDNA by electrostatic mode, the binding strength of the complex will be weakened in the presence of NaCl (Askari et al., 2019). The positive charge of Na^+^ will neutralize the negative charge of the phosphoric acid skeleton, which can affect the stability of the complex and change the absorbance. With the addition of NaCl, the absorbance of ctDNA and NAA-ctNDA complex hardly changed (Figure 2D), suggesting that Na^+^ strength did not interfere with the stability of the system and caused few obvious changes. These results indicated that electrostatic interaction was not the main way between NAA with ctDNA.

### Circular Dichroism Analysis

Circular dichroism spectroscopy (CD) measurement is an influential method to detect the structural changes of DNA. Electrostatic and groove binding modes have little or without impact on the CD peaks of DNA, but the intercalation binding modes can enhance both the positive and negative peaks and make the B-type structure of DNA more stable (Roy et al., 2018; Huang et al., 2019).

**Figure 3:**
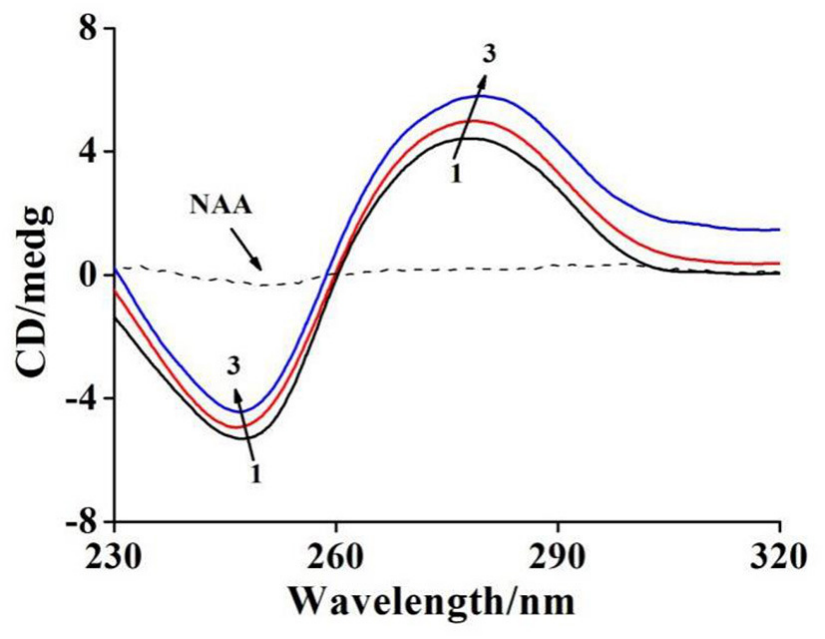
CD spectra of ctDNA with raising amounts of NAA at pH 7.4 and room temperature. *c*(ctDNA) = 6.44 × 10^−4^ mol L^−1^. The molar ratios of NAA to ctDNA were 0, 5/1, and 10/1 for curves 1 → 3, respectively.

The CD spectrum of ctDNA in B-form includes a negative peak (248 nm) and a positive peak (278 nm) due to the characteristic structures of right-handed helicity and stacked base-pairs (Sharifinia et al., 2020). As shown in Figure 3, The positive peak intensity of ctDNA intensified along with a certain degree of red shift (from 277 to 279 nm), while the negative peak intensity decreased accompanied by a slight blue shift (from 247 to 246 nm) after binding to NAA, suggesting that NAA had significant interference for the secondary structure of ctDNA, which also corresponded to the intercalation binding (Jaroslav et al., 2009). The variations of peak intensity in the CD spectra, in particular, indicated the stability of the right-handed B-form of ctDNA (Sharifinia et al., 2020), and the binding of NAA to ctDNA increased the base stacking degree of ctDNA and decreased the right-handed helicity of ctDNA (Li et al., 2014). Moreover, the red shift of the positive band demonstrated that ctDNA conformation tended to change from B-conformation to the more likely A-conformation as the π-π^*^ stacking of base pairs was weakened when NAA was inserted into ctDNA (Xia et al., 2017).

### Molecular Simulation

**Figure 4:**
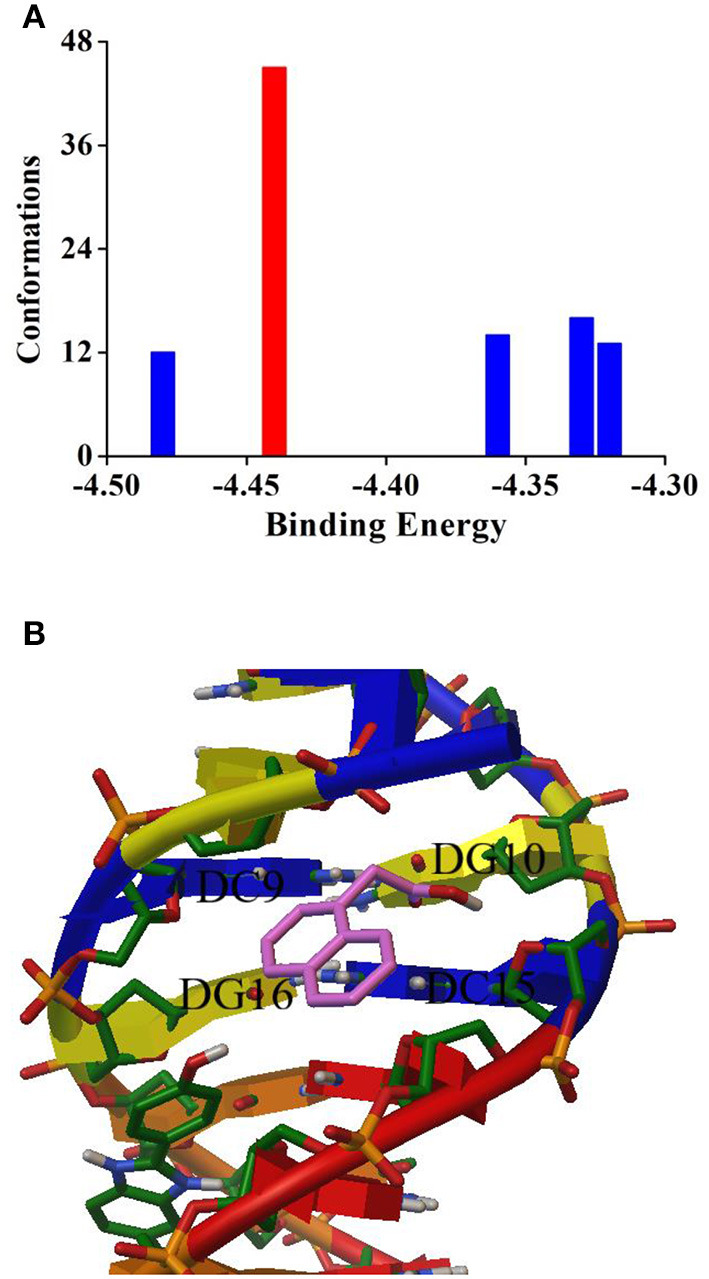
**(A)** Cluster analysis of the AutoDock docking runs between NAA with DNA. **(B)** Most possible docking pose of NAA with DNA.

To further verify the binding mode between NAA and ctDNA, the molecular simulation docking was applied to visually display the trial results. Based on the theorem of binding energy, 100 docking tests were carried out. As seen from Figure 4, the conformations with the highest number of binding times (45 times, −4.44 kcal mol^−1^) were obtained for final binding analysis (Figure 4A). It can be found that the lowest binding energy predicted by molecular simulation (−4.44 kcal mol^−1^ = −18.57 kJ mol^−1^) is slightly higher than that measured by thermodynamic experiments (−27.35 kJ mol^−1^), which may be due to the lack of desolvation energy in the vacuum environment of docking operation, similar results have been found in previous studies (Zhou et al., 2019). As seen from Figure 4B, NAA is bound with ctDNA in the groove region which is rich in G–C bases. Moreover, there is no hydrogen bond around it. This result confirmed the above trial results, namely, the binding mode of NAA and ctDNA was intercalation, and the main driving force was hydrophobic interaction.

## Conclusions

In this research, the interaction of NAA with ctDNA in a physiological buffer (pH 7.4) was investigated *in vitro* by various spectroscopic approaches, DNA viscosity measurement, melting point study, and molecular simulation. The results indicated that the fluorescence quenching of NAA by ctDNA was a mixed quenching process mainly consisting of the formation of the NAA–ctDNA complex. At 298 K, the binding constant of NAA and ctDNA was 0.60 × 10^5^ L mol^−1^, which suggested that the interaction between NAA and ctDNA was stable. Δ*S* > 0 and Δ*H* > 0 indicate that hydrophobic force is the main driving force of the reaction. The results of DNA melting point, the viscosity experiment, and KI quenching experiments show that the interaction mode of NAA and ctDNA is intercalation mode, while NaCl assay shows that there is no electrostatic force in the reaction. CD spectroscopy showed that NAA tended to induce changes in the secondary structure of ctDNA. Finally, molecular simulation results confirmed that NAA binds to ctDNA in an intercalation mode, and NAA can be found in the small grooves near the CG bases of ctDNA.

In recent years, with the improvement of people's living standards, people pay more attention to quality of life, and the requirements for the yield and quality of fruit and vegetable agricultural products have gradually improved. The application of plant hormones in modern agricultural production with high yield and quality is also gradually increasing. This study is conducive to raising people's awareness of NAA and strengthening the supervision and use of NAA by relevant departments.

## Data Availability Statement

The original contributions presented in the study are included in the article/supplementary material, further inquiries can be directed to the corresponding author/s.

## Author Contributions

XH: data curation, formal analysis, investigation, methodology, and writing—original draft. XL: investigation, methodology, formal analysis, and validation. ZZ: investigation and validation. RW: software and visualization. YH: project administration. GuiZ: data curation. GuoZ: conceptualization and writing—review and editing. All authors contributed to the article and approved the submitted version.

## Conflict of Interest

The authors declare that the research was conducted in the absence of any commercial or financial relationships that could be construed as a potential conflict of interest.
